# Veterinary communication can influence farmer Change Talk and can be modified following brief Motivational Interviewing training

**DOI:** 10.1371/journal.pone.0265586

**Published:** 2022-09-12

**Authors:** Alison M. Bard, David C. J. Main, Anne M. Haase, Helen R. Whay, Kristen K. Reyher

**Affiliations:** 1 University of Bristol Veterinary School, Langford, Bristol, United Kingdom; 2 Royal Agricultural University, Cirencester, Gloucestershire, United Kingdom; 3 Victoria University of Wellington, Faculty of Health, Wellington, New Zealand; 4 National University of Ireland Galway, Galway, Republic of Ireland; Brown University, UNITED STATES

## Abstract

Current veterinary communication skills training often focuses on the strategies necessary to successfully transfer information and promote shared decision making rather than inspiring client motivation to engage in behaviour change(s). One evidence-based communication methodology with a specific focus on enhancing conversations about change is Motivational Interviewing (MI), which is perceived by veterinarians to be highly relevant to their profession. We examined whether veterinarians who experienced brief (4–5 hours) MI training (BMIT) were able to change their communication behaviours to be more MI consistent. Fourteen veterinarians recorded 31 veterinary herd health consultations before (n = 15) and after (n = 16) BMIT to allow pre-post intervention analysis of veterinarian and farmer verbal behaviour. Additionally, using a sequential linguistic analysis of 3885 veterinarian-farmer communication events within these consultations, the influence of veterinarians’ verbal behaviours on farmers’ response language was explored. Analysis of veterinary consultations undertaken before and after BMIT revealed that veterinarians changed their communication style to be more consistent with the MI methodology, including more use of reflection statements, a more empathic and partnership-oriented consultation style and greater emphasis on clients’ own language in favour of change goals. In response, farmers contributed more to the conversation and discussed more herd health-related changes. Sequential linguistic analysis suggested that following a veterinarian emphasising something positive about the farmer (e.g. efforts, strengths), seeking collaboration or emphasising farmer choice, farmers were subsequently more likely to express arguments in favour of change (‘Change Talk’), especially phrases indicative of commitment. This study offers the first evidence of the potential value of a BMIT experience to enhance veterinary communication skills, although conscious and disciplined use of MI principles, strategies and Spirit–an ethos of compassion, acceptance, partnership and evocation—requires longer and more complex training. Further studies examining the longevity and consistency of these verbal behaviour changes following BMIT are required.

## Introduction

Inspiring farm clients to engage with behaviour change to improve herd health remains a critical challenge for the role of the cattle veterinarian [1]. The dairy industry is evolving worldwide, with attention shifting from the individual animal to management at the herd level, requiring a preventative and proactive advisory approach to veterinary work. To cope with these changes, veterinary herd health management (VHHM) programs have been established, broadly encompassing a combination of (advice on) animal health, milk production and disease prevention, placed in a framework of farm economics, welfare, food safety and environment [2]. Regular checks of herd and farm are important and often create communication opportunities within which VHHM advisory discourse can be situated, such as when carrying out routine fertility examinations of cattle [3].

To engage with VHHM advisory messages on change, farmers report the desire to be acknowledged for their competence and decision-making abilities on farm, to experience negotiation, collaboration and choice in herd health planning processes and to experience a trusting relationship with their veterinarian [3–5]. These desires reflect core attributes at the heart of Self-Determination Theory (SDT) [6], a macro-theory of human motivation. SDT identifies 3 innate psychological needs as critical to integrating externally recommended behaviours with an individual’s sense of self to stimulate internalised motivation: the need to feel competence (perceived self-efficacy), autonomy (sense of choice) and relatedness (connection with another). Meeting or thwarting SDT psychological needs underpins differences in whether farmers strive to extend their on-farm competencies or become uninterested and apathetic towards positive change [7].

The current herd health communication paradigm may often conflict with these psychological needs. Veterinarians tend to control herd health agendas, relying predominantly on giving information, persuading and questioning [8–10] with little evocation of farmer opinions, ideas and values through open questions [8, 11] all of which indicate a paternalistic communication approach. This mismatch between current and desired practice presents a barrier to client behaviour change, which may fundamentally undermine client engagement with veterinary herd health management (VHHM) activities. Indeed, at present, involvement in VHHM advisory discussion on specific VHHM topics does not seem to directly relate to improvement of farm performance parameters relating to these topics [12]. This is of concern as poor management of herd health is to the detriment of the health, welfare and productivity of the cattle involved which, in turn, is linked with adverse mental health and wellbeing outcomes for their farmers [13, 14]. To equip veterinarians to meet farmers’ psychologically pertinent desires for self-direction within VHHM services and thus improve engagement with messages on change, targeted communication training and education focused on veterinarians’ clinical communication competencies must be at the heart of future VHHM programmes.

One communication methodology described as a ‘fruitful marriage’ with SDT for behaviour change interventions [15] is Motivational Interviewing (MI), a collaborative conversation style that parallels and operationalises the tenets of SDT [16–19], developed in the medical sciences for strengthening a person’s own motivation to change [20]. Both SDT and MI have developed a common set of metatheoretical beliefs regarding positive human nature [15], viewing clients as growth-orientated with a natural tendency towards personal development. The task of an advisor is therefore viewed as to facilitate this natural change process by evoking and strengthening a client’s inner resourcefulness, rather than imposing or ‘installing’ motivation in a client from externally controlling strategies [15]. This similarity arguably underpins the congruence witnessed between the guiding principles of MI and the SDT psychological needs of relatedness, autonomy and competence [17].

MI is an evidence-based communication methodology for enhancing conversations about change, which specifically explores and resolves ambivalence to influence the motivational processes that facilitate change. Critical to this process is an interpersonal context of empathy, acceptance and partnership, with this relational foundation both a primary cause of improvement as well as a facilitator of positive client language surrounding change (The Relational Hypothesis: [21]. In turn, technical MI communication skills that shape and enhance this language further are adopted, with better MI skills predicted to increase the frequency and strength of client arguments in favour of change (‘Change Talk’), decrease client arguments in opposition to change (‘Sustain Talk’) and, in turn, positively influence behavioural outcomes (The Technical Hypothesis: [22]. Process research examining this ‘causal chain’ of effect offers strong empirical support for the link between MI skills and client response language [23], whilst the link between client response language and change outcomes is ‘promising’ [24]. Commitment Change Talk (referencing agreement, intention or obligation regarding behaviour change) has been framed as a key causal predictor of subsequent behaviour change within the Technical Hypothesis [22], suggested by Amrhein et al. [25] as indicative of clients that are approaching the resolution of ambivalence (conflicting ideas or emotions) on proposed change(s).

Existing research on MI within VHHM is in its infancy. Svensson et al. [26, 27] suggest MI is perceived by veterinarians to be highly relevant to their profession and that training can create meaningful shifts towards MI-consistent communication practice that are associated with increased client Change Talk. The exemplary design [28] of MI training underpinning these data– 6 days stretched over 7 months to enable coaching, feedback and self-directed learning—is, however, time intensive. Whilst more brief MI training can never claim to offer the opportunity to learn MI in all its nuance and depth [29], shorter MI training can still evidence meaningful outcomes [30] and is likely more suited to the time-limited nature of veterinary practice. Research examining whether more brief MI training significantly impacts veterinary communication is therefore imperative to support evidence-based dissemination of MI to cattle practitioners. Additionally, Svensson et al’s [27] examination of client outcomes did not capture immediate, within-consultation linguistic transitions during VHHM consultancies. This detail is critical to understand how specific within-session veterinary communication behaviours influence farmers’ Change Talk and Sustain Talk and their resulting commitment to change in discussions of herd health management [the MI ‘causal chain’; 20, 31].

To explore the provision of a brief MI training experience for improving communication skills of veterinarians and enhancing farmer engagement within VHHM consultancies, this study therefore examined 3 research questions. First, to understand whether brief MI training (BMIT) is sufficient to change veterinarians’ communication behaviour: 1) Does BMIT significantly impact the verbal behaviour of veterinarians and their farm clients when assessed using a pre-post intervention design?; Secondly, to explore the VHHM ‘causal chain’: 2) are specific veterinary verbal behaviours more or less likely than chance to lead to Change, Sustain or Neutral farmer response language than others? and 3) are specific veterinary verbal behaviours more or less likely than chance to lead to farmers articulating Commitment Change Talk (referencing agreement, intention or obligation regarding behaviour change)?

## Materials and methods

### Participants, training and study consultation data

One regional veterinary group (i.e. veterinarians from different practices located in the same UK region, who met regularly to discuss a special veterinary interest) and six veterinary practices (veterinarians n = 60) in the UK were recruited to take part in a study of BMIT, running from September 2016 to April 2017. Both the veterinary practices and regional group were a convenience sample, recruited by email, telephone or face-to-face interactions from practices or via individuals known to the research team and agreeing in principle to this study protocol. Participating practices received 2 training sessions on MI methodology via their ‘clinical clubs’ (lasting approximately 2 hours contact time per session), whilst the regional group of veterinarians received comparable training content delivered during a one-day meeting (approximately 5 hours contact time).

The training protocol was designed and delivered by author AB, who is a member of the Motivational Interviewing Network of Trainers (http://www.motivationalinterviewing.org/). BMIT was a mixture of didactic presentation and demonstration, dyadic or small group experiential exercises, video observation and group feedback on core elements of MI practice. Training was informed by established theory on motivational engagement and effective learning, adopting principles of Experiential Learning Theory [32] and SDT (6) in its design and delivery. Topics encountered included MI Spirit–the ethos of partnership, evocation, acceptance and compassion in MI communication interactions–the MI ‘core’ verbal skills of open questions, affirmations, reflections and summaries, the Four Processes within an MI conversation of Engage, Focus, Evoke and Plan and ‘core ideas’ of MI (giving MI-consistent advice, recognising and responding to client expression of Change Talk and Sustain Talk and the psychology of client ‘reactance’). Further information and clarification of these topics can be found in Miller and Rollnick [33].

To allow analysis of veterinarian and farmer discourse, all participating veterinarians were invited to record consultations with a farm client on *‘any change for the benefit of herd health’* (for example, when providing advice on lameness management) before and after their BMIT experience. Veterinarians recording consultations were provided with a handheld dictaphone and microphone for this purpose so that consultation data could be recorded without interrupting the typical veterinarian-client encounter (*i*.*e* microphones could be clipped to the collar and connected to a dictaphone, worn in an internal pocket) in addition to appropriate study information sheets and consent forms. Veterinarians were responsible for recruiting farmers of their choice from their client base. Not all vets participated in recording consultations with clients after BMIT, resulting in pre-post intervention data (n = 31 consultations) from a partial cohort (n = 14) of the total trained (n = 60) veterinary participants.

### Ethics

This study was reviewed and approved by the University of Bristol Faculty of Medical and Veterinary Science Research Ethics Committee (ref 14261) ensuring procedures met ethical guidelines in place for research with human participants. An information sheet was supplied to participants detailing the aims of the research prior to data collection, with written consent to take part obtained from both veterinarians and farmers involved.

### Verbal coding

#### Motivational Interviewing Treatment Integrity code

Veterinarian verbal behaviours were analysed using the MI Treatment Integrity 4.2.1 code [MITI; 34, 35] a treatment integrity measure for clinical trials of MI. Verbal interactions were coded for frequencies of (i) verbal behaviours and (ii) global scores [S1 Table]. For (i) verbal behaviours, discourse was broken down into volleys (uninterrupted segments of speech) and coded for utterances (complete thoughts) reflecting MITI verbal behaviours of Giving Information, Persuade, Persuade with Permission, Question, Reflection (both Simple and Complex), Affirm, Seek Collaboration, Emphasise Autonomy and Confront. MITI utterances identified as ‘Structuring Statements’ (those indicating what was going to happen during a consultation, instructions within a consultation such as *‘move that cow into the crush’* or information regarding set-up of another veterinary appointment) were also included in the coding process–as in sequential MI coding manuals [36] - to ensure all communication transitions would be accurately represented in sequential analysis of veterinarian-farmer verbal behaviour. Each utterance in a volley received only one behaviour code and each volley earned each code only once [28]. For (ii) global scores, the coder assigned a single number on a 5-point Likert scale (from 1: low proficiency to 5: high proficiency) to characterise the entire interaction, providing an overall judgement about the global interaction measures of Cultivating Change Talk, Softening Sustain Talk, Partnership and Empathy.

#### Client Language Assessment in Motivational Interviewing code

Farmer verbal behaviour was analysed using the Client Language Assessment in Motivational Interviewing (CLAMI) code [37] to capture frequency and type of client language about change. For verbal behaviours, discourse was broken down into volleys and coded for utterances reflecting Change Talk, Sustain Talk and Follow/Neutral [S2 Table]. Both Change Talk and Sustain Talk were further subcategorised as Reasons (subcodes Desire, Ability, Need), Taking Steps, Commitment and Other. CLAMI was coded according to MITI principles, where each utterance in a volley received only one behaviour code and each volley earned each code only once [34].

#### Study irrelevant coding

Sequences of MITI and CLAMI verbal behaviours were of interest in this study. As unique VHHM non-MITI and non-CLAMI behaviours also contributed to volleys of consultation dialogue, a selection of codes was developed to additionally capture all ‘study-irrelevant’ verbal behaviour, such as the veterinarian or farmer talking to the cow [S3 Table].

Final variables for analysis calculated from coding instrument data are indicated in Table 1. For pre-post intervention analysis using SPSS Version 23, all variables other than Change Talk subcodes (Reasons, Taking Steps, Commitment, Other) were included. For sequential linguistic analysis using GSEQ 5.1, veterinarian MITI behaviour frequency, farmer CLAMI behaviour frequency and farmer CLAMI summary measure variables were included.

**Table 1:** Final variables for inclusion in statistical analysis of herd health consultation (n = 31) data for (i) pre-post analysis of brief Motivational Interviewing training and (ii) the ‘causal chain’ of effect within veterinary herd health management consultations.

Coding		Final variable	Created by
VETERINARIAN	MITI Behaviour frequency	MI-adherent	Seek Collaboration + Emphasise Autonomy + Affirmation
		Reflection	Simple Reflection + Complex Reflection
		MI-inadherent	Persuade + Confront
		Question	Open Question + Closed Question
		Other	Giving Information + Persuade with Permission + Structuring Statements
	MITI Summary measure	Reflections per Question	(Simple Reflection + Complex Reflection) /Total Questions
		% Complex Reflections	Complex Reflection/(Simple Reflection + Complex Reflection)
		Relational	Empathy + Partnership/2
		Technical	Cultivating Change Talk+ Softening Sustain Talk/2
FARMER	CLAMI Behaviour frequency	Change Talk Reasons	[No alteration made to original variable]
		Change Talk Taking Steps	
		Change Talk Commitment	
		Change Talk Other	
		Follow/Neutral	
	CLAMI Summary measure	Sustain Talk (ST)	ST Reasons + ST Taking steps + ST Commitment + ST Other
		Change Talk (CT)	CT Reasons + CT Taking Steps + CT Commitment + CT Other
	MITI CLAMI Study-irrelevant	Farmer proportion of total consultation speech	All CLAMI + Farmer Irrelevant + Farmer Cannot Hear Content/ All CLAMI + Farmer Irrelevant + Farmer Cannot Hear Content + All MITI + Vet Irrelevant + Vet Cannot Hear Content
BOTH	Study-irrelevant	Study-irrelevant	Sum of all study-irrelevant codes: S3 Table

MI = Motivational Interviewing; MITI = Motivational Interviewing Treatment Integrity code [34]; CLAMI = Client Language Assessment in Motivational Interviewing code [37]; CT = Change Talk; ST = Sustain Talk.

### Data editing

Prior to verbal behaviour coding, pre and post- BMIT recorded consultations (n = 31) were assessed for length. Those consultations exceeding 20 minutes in length were edited to retain only the final 20 minutes of the consultation for 2 reasons: firstly, this is the maximum coding time window recommended within MITI due to diminished reliability of globals above this threshold (34). Secondly, sampling the last 20 minutes was proposed by Dr Lars Forsberg [2017, personal communication] of the coding laboratory MIC Lab Stockholm (www.miclab.se) to offer the opportunity to capture comparable consultation segments where change conversations were expected to peak as the consultation drew to a close and momentum towards planning and action occurred.

Following verbal behaviour coding, frequency data for those consultations less than 20 minutes in length were adjusted to reflect a 20-minute sample to ensure equitable and thus statistically sound comparisons of verbal behaviour frequencies before and after the MI training experience. For example, verbal behaviour frequencies in a consultation sample of 16 minutes would be multiplied by 1.25. For those veterinarians (n = 2) that submitted more than one consultation sample for pre- or post-training analysis, a mean was calculated from both consultation samples for MITI behaviour frequencies/globals and CLAMI behaviour frequencies.

### Data organising

To ensure coding was completed blind to treatment (pre- or post-training), consultation samples were pooled across treatments and a third party uninvolved in this research project (i) randomised the order of consultation samples using the RAND function in Microsoft Excel and (ii) renamed each audio (.wav) sample accordingly with the relevant anonymous code (1–31). Data were subsequently converted to movie format (.mp4) to allow them to be imported by anonymous code into Noldus Observer XT [38] an event-logging software for behavioural interaction analysis. To enable analysis within Noldus Observer XT, a coding scheme combining the MITI and CLAMI coding instruments in addition to ‘study irrelevant’ codes [S3 Table] was created, with each individual verbal behaviour set as a mutually exclusive state event. Durations of verbal behaviour were additionally of interest; therefore, extra codes were created to distinguish between the ‘first occurrence’ and a ‘repeat occurrence’ of a MITI of CLAMI verbal behaviour code in a veterinarian or farmer volley. This ensured that total durations of any given code could be accurately summarised through combining ‘first occurrence’ and ‘repeat occurrence’ verbal behaviour codes, whilst frequency counts reflecting MITI and CLAMI outputs—where only the first occurrence of a code in a volley contributed to the final tally—could be summarised by accounting for only ‘first occurrence’ behaviour codes.

### Coding process and coder

MITI global scores were completed on the first pass of the recording, whilst MITI and CLAMI verbal behaviour coding was completed with a second pass. This process was chosen as MITI and CLAMI recording required stopping and restarting of the recording during the coding process, which is reported to disrupt the ability of the coder to form a gestalt impression for the global codes [34]. This stopping and restarting was a necessary component of coding, given interest not only in the frequency of verbal behaviours but also in their respective durations. Durations could only be accurately calculated as an output with accurate parsing, which necessitated listening to volleys (or even sequences of volleys) in full prior to rewinding the recording and parsing accordingly. This process allowed careful identification of start and stop points for utterances in addition to differentiating ‘first occurrence’ and ‘repeat occurrence’ behaviours within volleys.

This combination of CLAMI [37] with MITI [34] analysis—as applied in other MI research interventions [39, 40] - is reported to be practical and achieve good reliability [39], with Klonek, Quera and Kauffield [41] highlighting the capacity for computer-supported coding to aid this process. However, the combination of CLAMI-, MITI-, and VHHM-specific codes within Noldus Observer for sequential analysis excluded the possibility of support from commercial coding laboratories in data analysis. All coding of verbal behaviours was therefore completed by author AB, who was blinded to treatment group (pre- or post-BMIT), following training in Motivational Interviewing coding (Moyers and Ernst, Cardiff: 2015), continuing professional development (e.g. via MINT) and personal coaching (author AH).

Coding consistency was assessed on an intracoder basis (analysis presented in S1 Text) to provide confidence in these data. An event-based Kappa coefficient calculated using GSEQ 5.1 [42] indicated strong intracoder agreement (K(E) = 0.81) within a sub-sample of randomly selected double-coded consultation files (n = 4, >10% observation time) indicating consistency in code attribution throughout the data analysis process. When performing a Spearman’s rank correlation (SPSS Statistics 23), both MITI [34] and CLAMI [37] codes also evidenced theoretically meaningful statistical associations, with Relational global scores positively correlated with Reflection use (*p<*0.0005) and Technical global scores positively correlated with Change Talk (*p* = 0.001*)* and negatively correlated with Sustain Talk (*p = 0*.*04)*.

### Statistical analysis

#### Pre-post intervention analysis of BMIT

Coding of verbal behaviour yielded a dataset that was unevenly distributed across MITI and CLAMI coding categories. Given the small sample size, MITI and CLAMI data were summarised into 12 variables to increase analytical power in accordance with the recommendations of Martin et al. [36] and Moyers et al. [34] for veterinarian verbal behaviour and Miller et al. [37] for farmer verbal behaviour (Table 1). One additional variable unique to this study, representing the proportion of consultation speech attributable to the farmer, was additionally calculated (Table 1). This resulted in a total of 13 variables for inclusion in statistical analysis to explore whether significant differences existed in verbal communication behaviours between pre- (n = 15) and post- (n = 16) BMIT intervention consultations using SPSS Version 23 (IBM Corp., Armonk, NY; Table 1). Non-parametric sign tests were conducted to compare veterinarian Summary Measures (Relational, Technical: Table 1: S1 Table) before and after BMIT. For all other veterinarian and farmer verbal behaviours (Table 1) paired sample t-tests were conducted to compare verbal behaviours before and after BMIT.

#### The VHHM ‘causal chain’

Sequential data analysis (using software GSEQ 5.1; 42) was performed using event sequential data (streams of text code representing veterinarian and farmer utterances as they unfolded linearly within the consultation interaction). In sequential coding, the relative temporal position of utterances is described as a ‘lag’, with the first utterance in a series of interest defined as lag 0, the second as lag 1, the third as lag 2, and so on. For example, in the exchange “(A) [*How is your lameness management*?] (B) [*I’m committed to scraping my sheds every day*] (C) [*But I guess struggle with foot trimming]*”, if utterance (A) is designated as the given veterinarian verbal behaviour in a herd health consultation at lag 0, utterance (B) is the lag 1 farmer behaviour, whilst utterance (C) is the lag 2 farmer behaviour. As in comparable studies [43] the primary measures of interest in this study were transition probabilities at lag 1; that is, estimations of the probability that the very next farmer verbal behaviour to occur, following a veterinarian verbal behaviour, would come from (for example) Change Talk, Sustain Talk or Neutral/Follow coding categories.

To have a sufficient sample size of behavioural transitions for analysis, data were pooled across all pre- and post- BMIT consultations (n = 31), as in similar studies [43, 44]. The data set yielded 3885 transition events between veterinarian (MITI) and farmer (CLAMI) verbal behaviour, which were unevenly distributed across 21 categories [S1 and S2 Tables]. Given the large number of transitions, data were initially explored to determine if these 21 individual codes could be sequentially analysed. However, to obtain reliable estimates of transition probability significance, Chi-square tests on contingency tables larger than 2x2 require no more than 20% of expected counts to be less than 5 and all individual expected counts to be one or greater [45]. A contingency table of all 21 MITI and CLAMI behaviours violated these assumptions.

To increase analytical power, veterinarian MITI behaviour codes were therefore collapsed across 5 coding categories in accordance with Martin et al’s [36] recommendations to create the veterinarian codes of MI-adherent, MI-nonadherent, Reflection, Question and Other (Table 1). Event sequential analysis was performed with these 5 veterinarian codes over lag 1 for veterinarian-farmer transitions using permutations of farmer response data, to be able to answer 2 research questions identified:

- Are specific veterinary verbal behaviours more or less likely than chance to lead to farmers articulating Change, Sustain or Neutral response language?
  *Data: Farmer Change Talk, Sustain Talk and Follow/Neutral* (Table 1)
- Are specific veterinary verbal behaviours more or less likely than chance to lead to farmers articulating Commitment Change Talk (referencing agreement, intention or obligation regarding behaviour change)?
  *Data: Farmer Change Talk sub-codes of Reason, Taking Steps, Commitment and Other* (Table 1)

These analytical strategies ensured test assumptions were met for both analyses [45].

## Results

### Participants and consultation data

Of those veterinarians who attended BMIT (n = 60), 14 participants engaged with the research protocol for pre-post intervention analysis (i.e. attendance at both clinical clubs or full day session as well as completion of pre- and post-BMIT recordings with appropriate consent forms submitted for both veterinarian and farmer(s)). These veterinarians provided further demographic data (Table 2). Veterinarians (n = 14) recorded at least 2 samples for analysis: one pre-BMIT and one post-BMIT consultation. Two veterinarians submitted additional samples: one an additional pre- BMIT sample only, one additional pre- and post-BMIT samples. This created a total of 31 consultations for analysis. Two consultations involved the participation of one veterinarian and 2 farm clients; as in previous sequential analyses [46], CLAMI coding of these consultations included contributions from both farm clients involved in the interaction.

**Table 2:** Demographics study participants (n = 14) with completion of full study protocol.

Demographic	Veterinarians
**Gender**	Male (4), Female (10)
**Age**	21–30 (5), 31–40 (6), 41–50 (2), 60+ (1)
**Years in practice as a veterinarian**	1–5 (4), 6–10 (4), 11–15 (3), 16–20 (1), 21+ (2)

Average consultation sample length was 23.3 minutes (range 4.7–74.4), with consultation topics representing calf health (n = 9), herd health (>1 topic area: n = 6), mastitis (n = 6), lameness (n = 3), Johne’s (n = 2), fertility/breeding (n = 2), heifer health and welfare (n = 1), dry cow health and welfare (n = 1) and nutrition (n = 1). Topics covered within these consultations were diverse [S4 Table]. Consultation samples were submitted within 5 weeks of the completed BMIT experience.

### Pre-post intervention analysis of BMIT

Full participant data are summarised in S5 Table. When assessed purely through Relational and Technical summary measures, one veterinarian achieved a level of ‘fair’ competence pre-BMIT according to MITI [34] guidelines (Relational ≥ 3.5, Technical ≥ 3), whilst 8 achieved ‘fair’ competence post-BMIT. Assessing MI competency across all MITI summary measures, none achieved a ‘fair’ level of competence pre-BMIT according to MITI [34] guidelines (Relational ≥ 3.5, Technical ≥ 3, ≥ 40% Complex Reflection, ≥ 1:1 Reflection to Question ratio), whilst 3 achieved ‘fair’ competence post-BMIT.

**Fig 1:**
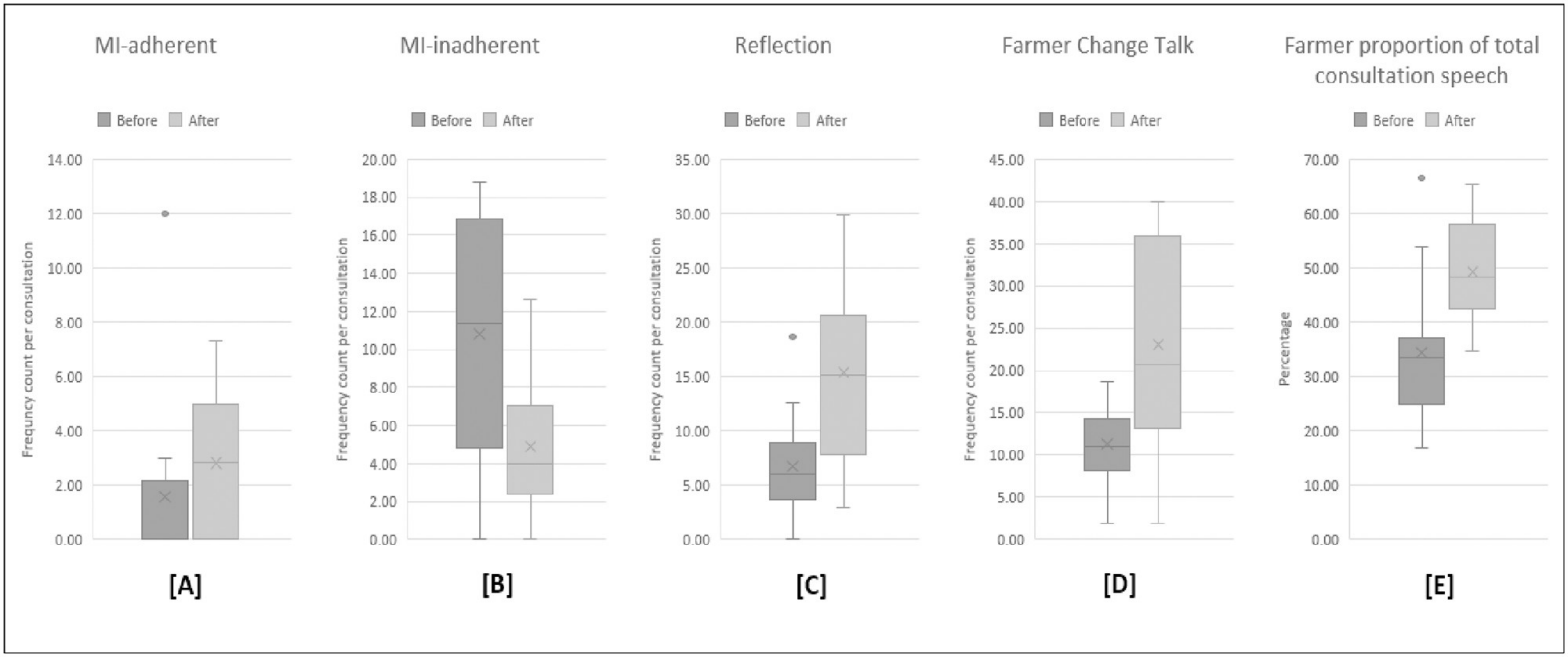
The interquartile ranges (shaded boxes), medians (middle solid line in shaded box), range of observed data (whiskers) and outliers (dots) of verbal behaviours before and after brief Motivational Interviewing training. (A) Emphasise Autonomy + Affirm + Seek Collaboration; (B) Persuade + Confront; and (C) Complex Reflection + Simple Reflection, in addition to (D) farmer frequency of Change Talk (Reasons + Taking steps + Commitment + Other) and (E) farmer proportion of total consultation speech (All Client Language Assessment in Motivational Interviewing (CLAMI) behaviour durations + Farmer Irrelevant durations + Farmer Cannot Hear Content durations/ All CLAMI durations + Farmer Irrelevant durations+ Farmer Cannot Hear Content durations + All Motivational Interviewing Treatment Integrity (MITI) behaviour durations + Vet Irrelevant durations + Vet Cannot Hear Content durations).

**Fig 2:**
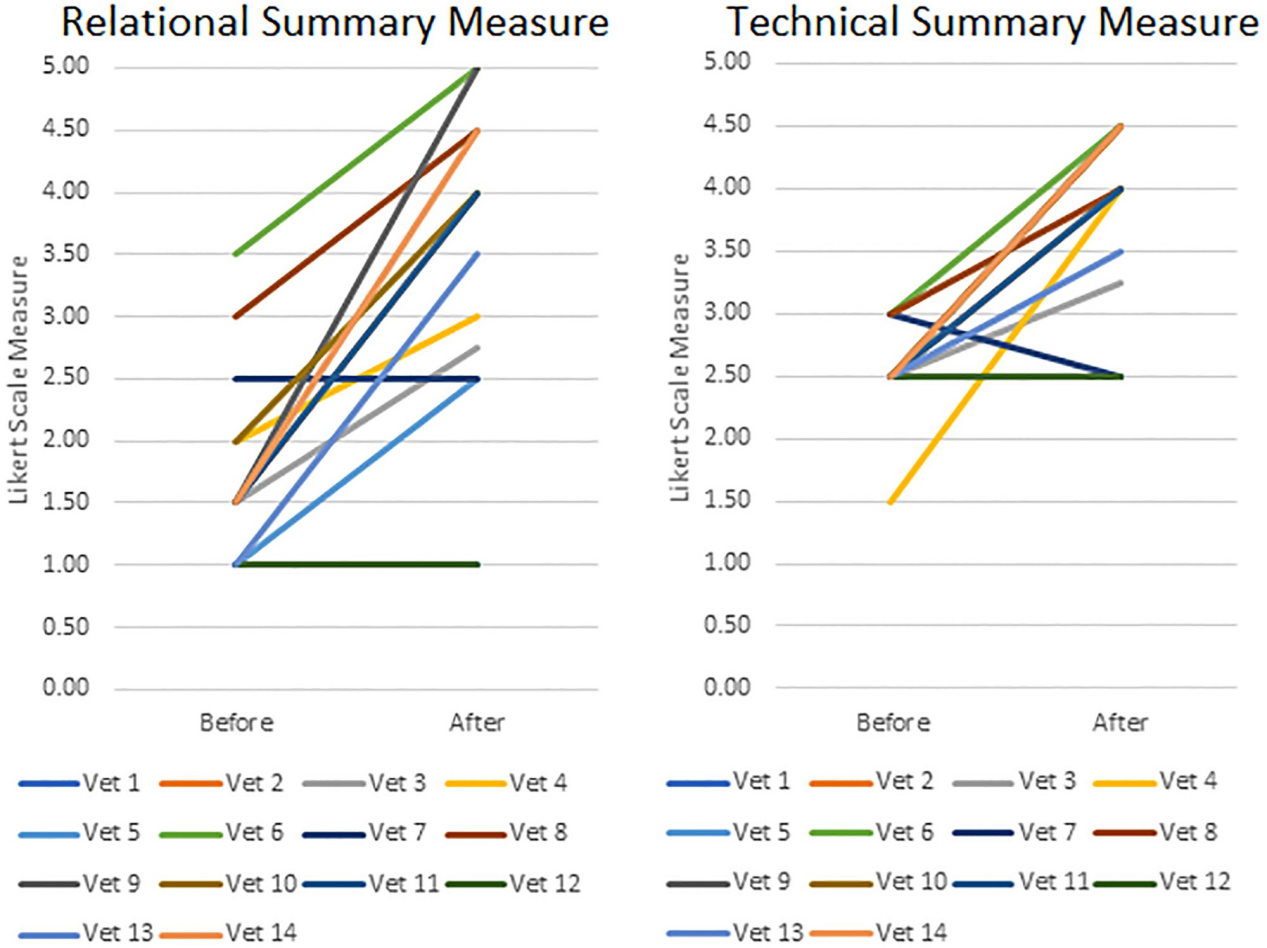
Line graphs indicating differences in veterinarian consultation (n = 14) summary measures before and after brief Motivational Interviewing training. (A) Relational (Empathy + Partnership/2) and (B) Technical (Cultivating Change Talk + Softening Sustain Talk/2).

Paired sample t-tests indicated a significant post-training increase in veterinarians’ use of the MI-consistent verbal behaviour Reflection (*p* = 0.01) and the number of these Reflections used per Question asked (*p* = 0.04), in combination with a significant decrease in use of MI-inconsistent behaviours Persuade and Confront (*p* = 0.001; Fig 1A–1C). A shift towards greater congruence with MI was also witnessed in veterinarians’ Relational and Technical summary measures, with non-parametric sign tests indicating a statistically significant median increase in veterinarians’ post-training Relational (Partnership and Empathy) scores (*p* = 0.001; Fig 2) and Technical (Cultivating Change talk and Softening Sustain Talk) scores (*p* = 0.01; Fig 2). Other veterinarian verbal behaviours were also significantly reduced in post-training consultations (*p* = 0.04).

Paired sample t-tests indicated farmers significantly increased their arguments in favour of change for herd health (Change Talk; *p* = 0.02) in post-training consultations and also contributed a significantly higher proportion of total consultation speech (*p* = 0.01; Fig 1D–1E).

### The VHHM ‘causal chain’

#### Relationships between veterinary verbal behaviours and farmer response language

To test whether specific veterinary verbal behaviours were more or less likely than chance to lead to farmer Change, Sustain or Follow/Neutral response language, lag-sequence matrices were generated in GSEQ with veterinarian verbal behaviours (i.e., initial behaviours at lag 0) in rows and farmer verbal responses (i.e., subsequent behaviours at lag 1) in columns of the matrix. Table 3 presents these veterinarian-farmer transitions (i.e. veterinarian behaviour at lag 0 to the immediate farmer response at lag 1). The results show a significant relationship between veterinary verbal behaviours and farmer response language (*p* = <0.01), indicating a non-random pattern in veterinarian-client interactions.

**Table 3:** Adjusted residuals for farmer verbal behaviour in response to veterinarian verbal behaviour at lag 1 in pooled herd health consultation (n = 31) data.

	Subsequent behaviour at lag 1 (farmer)		
Initial behaviour at lag 0 (veterinarian)	Change Talk	Follow/Neutral	Sustain Talk
MI-adherent *Affirm*, *Seek Collaboration*, *Emphasise Autonomy*	4.83**	-4.04**	-0.88
Reflection *Simple and Complex*	5.32**	-6.18**	2.33*
MI-inadherent *Persuade*, *Confront*	3.78**	-5.34**	3.47**
Question *Open and Closed*	-4.97**	5.83**	-2.29*
Other *Give Information*, *Persuade with Permission*, *Structuring Statements*	-2.73*	3.12**	-1.09

χ2 (8) = 104.34 (*p* = 0 < .01)

*p<0.05,

** p<0.01

MI = Motivational Interviewing

Subsequently, adjusted residuals were calculated to determine the strength of the specific associations between each veterinarian behaviour (e.g. Reflection) and subsequent farmer behaviour (e.g. Change Talk). Adjusted residuals are standardized raw residuals (based on the difference between the observed and expected frequency). This cell-specific statistic indicates whether a sequential association between a veterinarian behaviour at lag 0 and a subsequent farmer response at lag 1 is significantly more or less likely than expected by chance [42].

**Fig 3:**
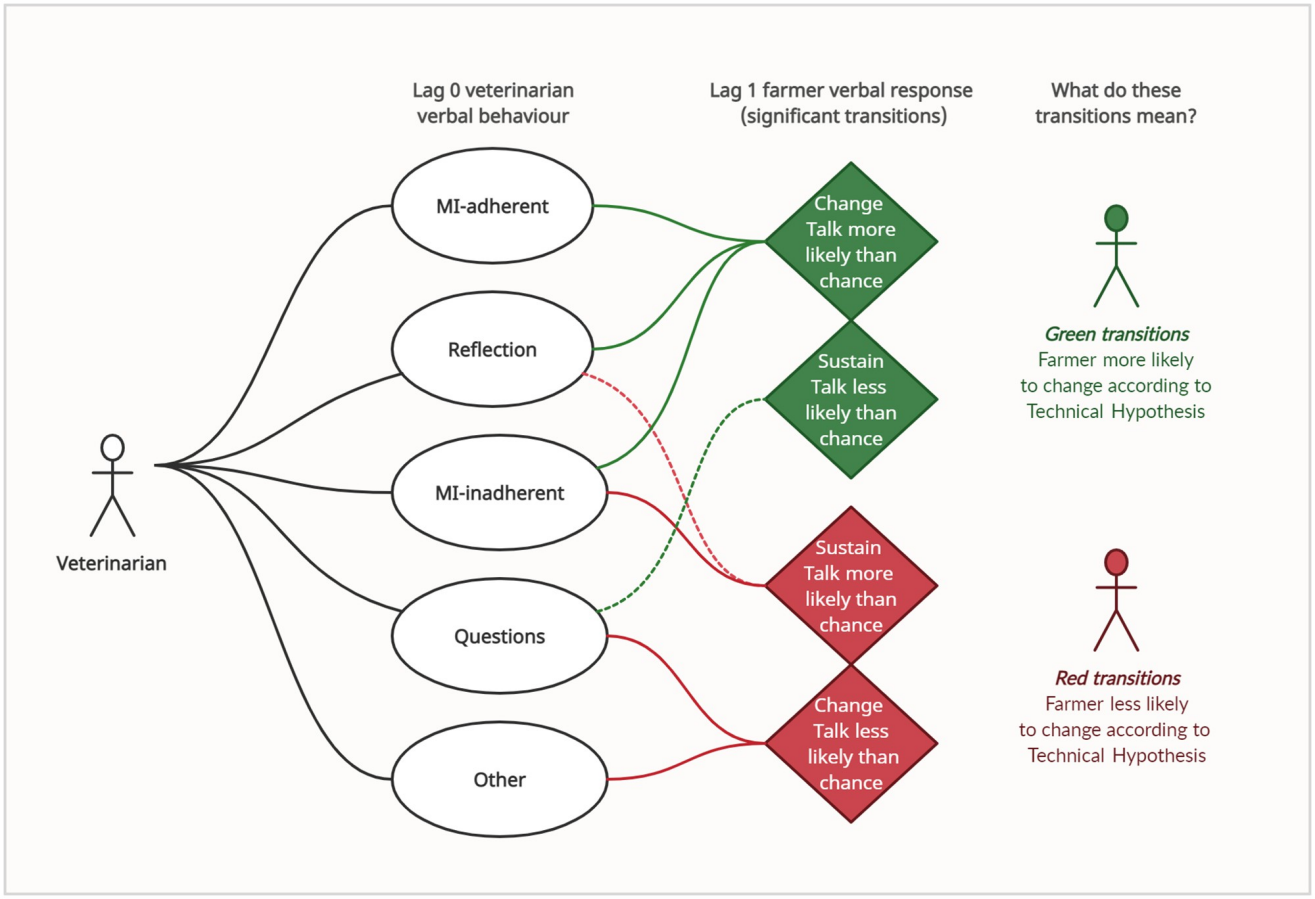
Significant transitions between veterinarian verbal behaviour (lag 0) and farmer verbal response (lag 1) in pooled herd health consultation (n = 31) data, with hypothesised effect on farmer change outcomes according to the Technical Hypothesis of Motivational Interviewing. MI = Motivational Interviewing; Solid green/red lines: *p<0*.*01;* Dotted green/red lines: *p<0*.*05*.

**Fig 4:**
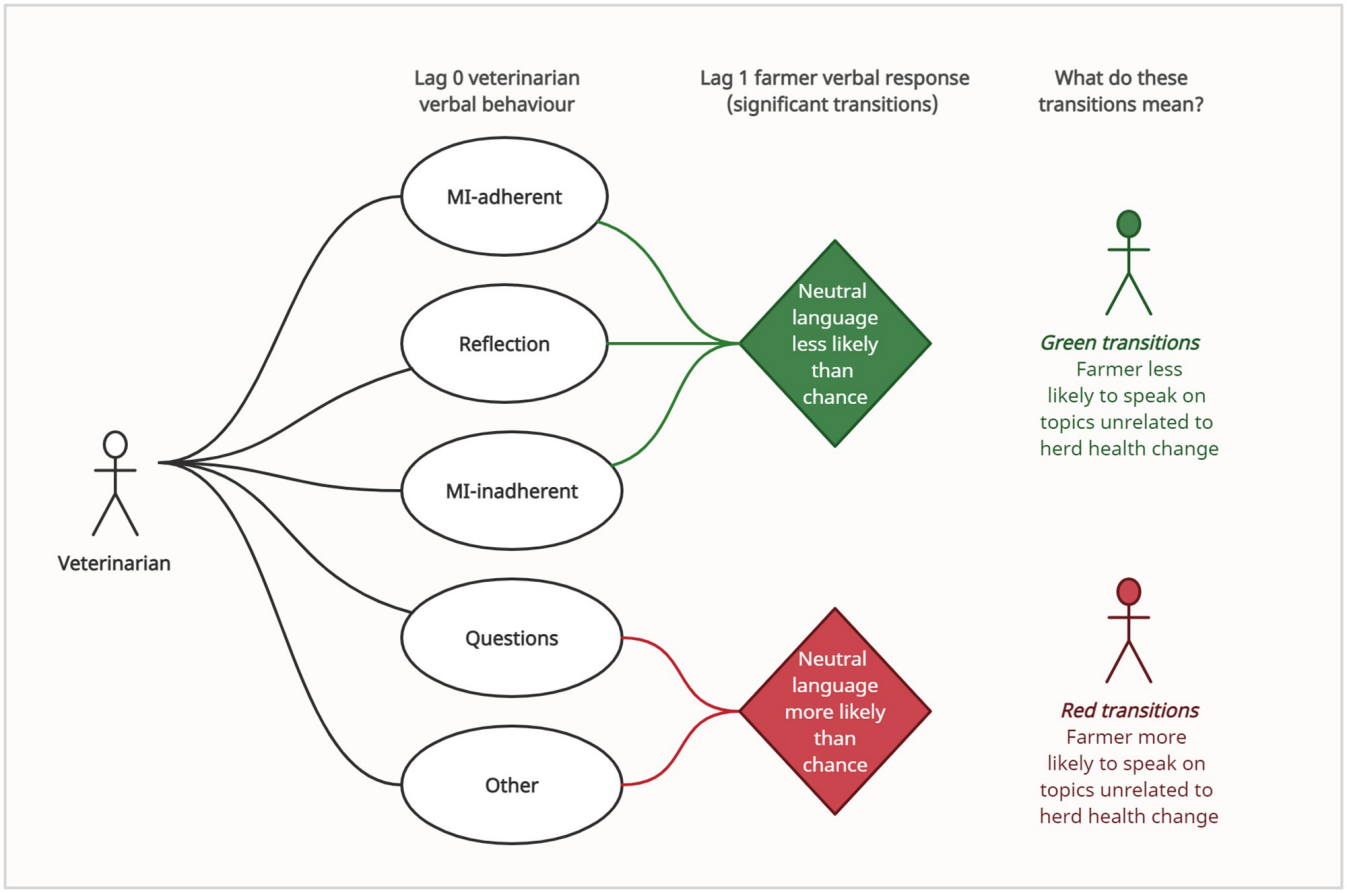
Significant transitions between veterinarian verbal behaviour (lag 0) and farmer response (lag 1) in pooled herd health consultation (n = 31) data and their effect on farmer vocalisation of herd health change topics. MI = Motivational Interviewing; Solid green/red lines: *p<0*.*01*.

Change Talk followed MI-adherent (*p*<0.01), Reflection (*p*<0.01) and MI-inadherent (*p*<0.01) verbal behaviours more frequently and Question (*p*<0.01) and Other (*p*<0.05) behaviours less frequently than expected by chance. Sustain Talk followed Reflection (*p*<0.01) and MI-inadherent (*p*<0.05) behaviours more frequently and Question (*p*<0.05) less frequently than expected by chance. Follow/Neutral behaviours followed Question and Other behaviours more frequently and MI-adherent, Reflection and MI-inadherent less frequently than expected by chance (*p*<0.01). Significant transition probabilities between lag 0 veterinarian verbal behaviour and lag 1 farmer response language, in addition to comment on the meaning of these significant transitions, are presented in Fig 3 (farmer Change Talk and Sustain Talk) and Fig 4 (farmer Follow/Neutral).

#### Relationships between veterinarian verbal behaviours and farmer Commitment Change Talk

To test whether specific veterinary verbal behaviours were more or less likely than chance to lead to farmer Commitment Change Talk, lag-sequence matrices were generated in GSEQ with veterinarian behaviours in rows (i.e., initial behaviours at lag 0) and farmer Change Talk–Reasons, Desire, Commitment, Other- in columns of the matrix (i.e., subsequent farmer response at lag 1). Table 4 presents these veterinarian-farmer transitions (i.e. veterinarian behaviour at lag 0 to the immediate farmer Change Talk at lag 1). The results again showed a significant relationship between veterinary verbal behaviours and farmer response language (*p* = 0.01), indicating a non-random pattern in veterinarian-client interactions. Subsequently, adjusted residuals were calculated to determine the strength of the specific associations between a veterinarian behaviour (e.g. Reflection) and subsequent farmer Change Talk (e.g. Reasons). Commitment Change Talk followed MI-adherent (*p*<0.01) verbal behaviours more frequently than expected by chance. Reasons Change Talk followed Reflection (*p*<0.05) verbal behaviour more frequently and MI-adherent (*p*<0.05) verbal behaviour less frequently than expected by chance.

**Table 4:** Adjusted residuals for farmer Change Talk behaviour in response to veterinarian verbal behaviour at lag 1 in pooled herd health consultation (n = 31) data.

	Subsequent event at lag 1			
Initial event at lag 0	Commitment Change Talk	Other Change Talk	Reasons Change Talk	Taking Steps Change Talk
MI-adherent *Affirm*, *Seek Collaboration*, *Emphasise Autonomy*	3.51**	-0.24	-2.19 *	0.57
Reflection *Simple and Complex*	-1.78	-1.47	2.49*	0.04
MI-inadherent *Persuade*, *Confront*	0.33	1.35	-1.14	-0.57
Question *Open and Closed*	0.75	-1.25	0.44	0.55
Other *Give Information*, *Persuade with Permission*, *Structuring Statements*	-1.34	-1.68	-0.60	-0.46

*Notes*: χ2 (12) = 25.86 (*p* = 0.01)

*p<0.05,

** p<0.01

MI = Motivational Interviewing

## Discussion

### Pre-post intervention analysis of BMIT

Following BMIT, veterinarians significantly increased verbal behaviours consistent with MI (Reflection, Reflections per Question) and significantly decreased their use of verbal behaviours inconsistent with MI (MI-inadherent: Persuade and Confront), suggesting a more empathic and evocative communication style within post BMIT consultations. A significant post-BMIT increase in veterinarians’ Technical summary measure suggested veterinarians focused more on evoking farmers’ own arguments in favour of change, whilst a significant post-BMIT increase in veterinarians’ Relational summary measure suggested veterinarians were better able to foster collaboration and power-sharing and made more explicit verbal efforts to grasp client perspective, experience and emotion [34]. In combination with the proportion of veterinarians achieving ‘fair’ competence in Technical and Relational summary measures [34] increasing from 7% pre-BMIT to 57% post-BMIT, and ‘fair’ competence in all MITI summary measures [34] increasing from 0% pre-BMIT to 21% post-BMIT, these data suggest that veterinarians made important changes towards adopting a more MI-consistent consultation approach post-BMIT. It is important to note that no significant shift in MI-adherent (Seek Collaboration, Emphasise Autonomy, Affirm) veterinarian verbal behaviour was witnessed post-BMIT; these behaviours are regarded as particularly critical to the MI methodology. MI is not ‘easy to learn’ [29] and brief training alone may simply not be sufficient for a shift in these more complex verbal behaviours to occur.

In the only other published study on training veterinarians in MI [26], Swedish cattle veterinarians (n = 38) received 6 full days of training stretched over 7 months to enable coaching, feedback and self-directed learning. Of these veterinarians, 6% reached overall ‘fair competency’ in summary measures after training according to the classification specified by Moyers et al. [34] and significant improvements in all MITI variables other than Seeking Collaboration, Simple Reflections and Confront were witnessed. Given the great contrast in training contact time and support, outcomes via BMIT were surprising. We hypothesise that the significant improvement observed in our intervention may have been influenced by the fact that: (i) participants submitting audio recordings (n = 14) were likely a self-selecting sample of veterinarians who were most interested in and committed to enhancing their communication skills in the pursuit of change from the total pilot population who attended BMIT (n = 60); (ii) where baseline veterinary communication was indicative of a directive paradigm [S5 Table]—a stark contrast to the guiding and evocative style of MI–even brief training may have offered ample novel insight for a measurable shift in communication approach; and (iii) the ethos of MI practice may have strongly resonated with this UK training cohort given its match with the idealised relational values embedded in the UK VHHM paradigm (3), supporting comparatively rapid skills adoption in many of the veterinary participants.

In post-BMIT consultations, farmers also significantly increased their arguments in favour of change for herd health (Change Talk). Hypotheses on the mechanisms of MI efficacy suggest the cause of this may be twofold: (i) enhanced veterinarian Relational communication skill (Empathy and Partnership) in post-training consultations may have acted as a facilitative condition for the spontaneous expression of Change Talk (the Relational Hypothesis) combined with (ii) better veterinarian Technical communication skill (Cultivating Change Talk, Softening Sustain Talk) to recognise, strengthen and evoke Change Talk when it occurred (the Technical Hypothesis; [20]). Farmers additionally contributed a significantly higher proportion of total consultation speech in post-training consultations. Combined, these data suggest a more MI-consistent consultation approach by veterinarians may be one way to increase positive farmer engagement in VHHM discussions and facilitate farmer motivation for positive change in matters of herd health.

### The VHHM ‘causal chain’

When veterinarians used MI-adherent behaviours in behaviour change consultations (Emphasising Autonomy, Seeking Collaboration, Affirming), there was a significant increase in the probability that the farmer would next discuss their reasons, ability, desire or needs in favour of making a change for herd health, steps that could be taken and their commitment or other thoughts for making a change (Change Talk). There was no significant increase in the probability of any other farmer verbal responses in response to MI-adherent (Affirm, Emphasise Autonomy, Seek Collaboration) behaviour. These findings are congruent with farmer reports of better engagement and implementation when a sense of collaboration, behavioural choice and relational trust are present in VHHM services [3–5] and with meta-analyses suggesting that MI-consistent verbal behaviour predictably increases both the strength and frequency of client Change Talk in diverse disciplines [22, 47]. Data suggest that in the context of VHHM, communication with an emphasis on farmer choice, a collaborative enterprise and articulating genuine recognition of farmers’ strengths, skills and accomplishments may help inspire farmers to engage proactively in discussions of herd health management behaviour change.

In accordance with wider literature [22, 47], the use of MI-inadherent verbal behaviour (Persuade, Confront) led to a significant increase in the probability that the farmer would next express Sustain Talk and verbalise their reasons, inability, desire or needs in opposition to change for herd health, steps against change or their commitment or other thoughts for not changing. However, MI-inadherent verbal behaviour by veterinarians also led to a significant increase in the probability of Change Talk from farm clients. This was unexpected, given the well-recognised psychological phenomenon of ‘reactance’—when we feel our psychological needs for autonomy and behavioural freedom are being imposed upon, we become motivationally aroused to re-establish them, a process that can be cognitively operationalised through counter-arguing [48]. As such, Sustain Talk is viewed as an interpersonal phenomenon in MI, hypothesised to emerge from persuasive and confrontational clinician-client interaction rather than mere client pathology [33].

The observed relationship between MI-inadherent behaviour and Change Talk may be explained by the relational setting underpinning UK veterinary interactions. Given the sampling methodology of this study–where veterinarians were invited to submit their own choice of consultations—it is reasonable to assume that those clients with whom veterinarians had the most positive working relationship would be most likely to be asked for (and to agree to) recruitment. Previous research [49] observed MI-inadherent skills to have an unexpected positive relationship with enhanced client involvement ‘*when*, *and only when’* specific interpersonal skills of the advisor convey a genuine and authentic stance, encouraging clients to interpret MI-inadherent skills as honest in their intentions rather than as an afront to the relationship. Positive working relationships between UK veterinarians and farmers are reported to be based specifically on the perception of honesty and authenticity [3], which may have similarly acted to attenuate the effect of MI-inadherent behaviour on psychological reactance in this sample. Of note, MI-inadherent coding reflected predominantly Persuade (99%) behaviour, arguably engendering less reactance potential than Confront (1%) [S1 Table]. Further research with multiple consultation samples per veterinarian, representing diverse relational ties and demographics, is needed to explore whether veterinarian-farmer working relationships, or another contributing factor (e.g. gender, social status, professional roles) underpins this temporal relationship. In lieu of this evidence, the significant transitional relationship between MI-adherent behaviour and Sustain Talk suggests advisors take a risk in using Persuade and Confront when communicating on change, given the well-established negative effect path between Sustain Talk and client change outcomes [22, 50].

The verbal behaviour of Reflection by veterinarians significantly increased (*p*<0.01) the probability that Change Talk or Sustain Talk would follow veterinarian statements whilst significantly (*p*<0.01) reducing the probability of Follow/Neutral response language (i.e. unrelated to herd health change) from farm clients. As with MI-inadherent behaviour, this significant transitional relationship with both Change Talk and Sustain Talk may suggest Reflection is a risky component in the behaviour change consultation, given the negative path between Sustain Talk and behavioural outcomes [22, 47, 50]. However, Reflections are both empathic and evocative in nature, with their use able to reinforce and strengthen client statements; the content and valence of a given Reflection is liable to elicit very different responses [33]. For example, reflecting a farmer’s reasons to stop mobility scoring is likely to engender a different response (Sustain Talk) than reflecting a farmer’s motivation to aim for fewer lame cows (Change Talk), whilst either manifests empathy on behalf of the speaker. In consequence, veterinarians trained in MI can use Reflection in a technical manner informed by MI Spirit [33], purposefully guiding this transitional relationship with Change Talk and Sustain Talk to support client engagement, enrich empathic understanding and shape client response language as appropriate throughout the consultation.

Interestingly, in the Reflection data, there was a significant increase in the probability of Change Talk or Sustain Talk following a veterinarian Reflection, in combination with a significant decrease in the probability of Follow/Neutral farmer response language (i.e. unrelated to herd health change) following veterinarian Reflection. Veterinarian consultation samples in this study predominantly represented performance below ‘fair’ MI competence established in MITI guidelines (Relational ≥ 3.5, Technical ≥ 3, ≥ 40% Complex Reflection, ≥ 1:1 Reflection to Question ratio; [34]) suggesting veterinarians were unlikely to be sufficiently skilled in MI to use Reflections consistently in a strategic manner to evoke Change Talk and soften Sustain Talk. Rather, then, these data support the relational hypothesis of MI driving this observed relationship: given a context of accurate and empathic listening, clients are more likely to spontaneously vocalise and explore change [21]. Veterinarians in VHHM services could therefore benefit from the deliberate adoption of the skill of Reflection in the pursuit of active, accurate empathic understanding of their clients, to encourage (more) open and transparent communication from clients in return regarding their thoughts on advised VHHM change.

The enhancement of client commitment to change and the resolution of ambivalence is central to the efficacy of MI [33]. Accordingly, the use of Commitment Change Talk (referencing agreement, intention or obligation regarding change) is framed as a key causal predictor of subsequent behaviour change within the Technical Hypothesis of MI [22], supported by empirical evidence [25]. For farm clients, the probability of this Commitment Change Talk occurring—such as *‘I’m going to start pre-dipping in the parlour’*—showed a significant increase only in response to the MI-adherent verbal behaviours of Emphasise Autonomy, Seek Collaboration and Affirm. These data suggest that MI communication is well positioned within the VHHM encounter to promote positive discussions of behaviour change and may also be critical to outcomes [25]. Given that, by design, MITI-Change Talk analysis necessitated the assessment of a smaller number of transition frequencies (n = 291) than MITI-CLAMI, studies with greater sample sizes are needed to verify this relationship in the VHHM paradigm.

### Interpreting this study

VHHM consultation samples were submitted at the discretion of participating veterinarians representing ‘*any change for the benefit of herd health’*. The complexity of management topics within VHHM were assumed to have the potential to stimulate discourse in which farmers felt ambivalence (conflicting ideas or emotions) about proposed change(s) and that the expression of Change Talk and Sustain Talk would be likely to emerge [33]. Data in this study supported this assertion. However, establishing client ambivalence was not a pre-requisite to recording, therefore varying client presentations may have influenced absolute verbal frequencies of Change Talk and Sustain Talk within these data; clients high in ambivalence would likely have offered more Change Talk and Sustain Talk than comparably non-ambivalent peers, potentially shaping veterinarian-farmer temporal transitions or BMIT pre-post summary measures. Future research, able to record detailed data on client baseline ambivalence within a larger study sample, are needed to explore this nuance within VHHM consultation presentations.

It is important to recognise that the coding of this study was completed by one individual (author AB), given the unique combination of CLAMI-, MITI-, and VHHM-specific codes within Noldus Observer for sequential analysis excluded the possibility of support from commercial coding laboratories in data analysis. This study presents a thorough intracoder assessment [S1 Text] which, in addition to author AB’s experience (see ‘Coding process and Coder’), guaranteed high quality in data assessment. Studies with multiple coders assessed for intercoder agreement at regular intervals would be the gold standard for an intervention of this kind, however, ensuring any individual coder bias that may occur even when faced with blinded samples is identified (e.g. individual coders being more or less likely to respond to attempts made to apply MI in consulting).

With regards to pre-post intervention analysis of BMIT, veterinarians were only required to submit one sample before and after their training experience, therefore further research where participating veterinarians record interactions with multiple (e.g., n≥5) clients before and after BMIT is needed to control for potential within-veterinarian heterogeneity [51]. Additionally, a larger sample size of both veterinarians and farm clients would offer greater confidence in the outcomes of this BMIT intervention. Intervention data also reflect veterinarians’ capacity to learn and employ MI communication skills in a short time frame (≤5 weeks) rather than the sustainability of these skills in practice. Where enhancement of skills from a single workshop is likely to decay without additional training enrichment such as coaching and feedback [24], measures of skill use at repeated intervals post-training would offer critical insight into the longevity of these communication changes from BMIT. However, this study indicates that for a group of engaged veterinarians, a brief experience of MI training may be sufficient to modify verbal behaviours within VHHM communication. The overall shift towards a more evocative, empathic and collaborative consultation style was likely to be an important improvement, where the Technical Hypothesis of MI emphasises the positive impact of enhanced MI communication skills on client response language and associated behaviour change outcomes [22]. However, no effect on MI-adherent behaviour was witnessed as a result of BMIT. These verbal behaviours are central to MI practice [33] whilst also being the only behaviours in sequential analysis that resulted in farmer Commitment Change Language—framed as a key causal predictor of subsequent behaviour change within the Technical Hypothesis [22]- at lag 1 more frequently than expected by chance.

Sequential analysis must also be viewed in light of the study limitations. The comparably limited veterinarian use of MI-adherent verbal behaviour compared to other veterinarian codes, along with the comparably limited farmer expression of Sustain Talk compared to other farmer codes, may have made it less possible to identify associations when examining MITI-CLAMI temporal relationships. Future research using larger sample sizes may identify further associations between veterinarians’ MI skills and client response talk that can add to and inform the observations of this study, in addition to allowing the examination of broader social, structural and demographic factors that may influence these discourse interactions (e.g. the gender concordance of relational pairings: [11]). However, insight into sequential patterns of VHHM discourse provided by data in this study echo well documented transitional effects in wider professional contexts [50], suggesting this examination of consultation data is likely to offer valid insights to veterinarians in the VHHM paradigm.

## Conclusions

Sequential linguistic analysis of 31 herd health consultations suggests farmer motivation towards change may be enhanced using an MI-consistent communication style. Within VHHM consultations, when veterinarians used MI-adherent verbal behaviours of Emphasising Autonomy, Seeking Collaboration or Affirming the farmer, farmers were subsequently more likely to express Change Talk, especially phrases indicative of commitment to change. Additionally, analysis of veterinary consultations undertaken before and after brief (4–5 hours) MI training revealed that the experience encouraged veterinarians to modify their communication style to include more MI-consistent behaviours. Veterinarians increased their reflections of farmer statements, used a more empathic and partnership-oriented consultation style and placed greater emphasis on farmers’ own language in favour of change goals. In response, farmers contributed more to the conversation and discussed more herd health-related changes. This study offers the first evidence that engaging veterinarians with MI through brief (4–5 hours) training may support their adoption of communication behaviours consistent with the MI methodology. Further studies examining the longevity and consistency of these verbal behaviour changes following brief MI training are required, as the MI evidence base currently indicates conscious and disciplined use of MI communication principles, strategies and Spirit requires longer and more complex training experiences.

## Supporting information

S1 Table A brief description of the 10 verbal behaviours, 4 globals and 6 summary measurements used in the assessment of Motivational Interviewing skills (Moyers et al., 2014). (DOCX)

S2 Table A brief description of the Change Talk, Sustain Talk and Follow/Neutral response language captured through Client Language Assessment in Motivational Interviewing coding system (Miller et al., 2008). (DOCX)

S3 Table ‘Study-irrelevant’ verbal behaviour codes constructed for analysis of veterinary herd health consultations. (DOCX)

S4 Table Consultations (n = 31) submitted by veterinarians (n = 14) representing a ‘change for the benefit of herd health’ either before or after a Motivational Interviewing training experience. (DOCX)

S5 Table A summary of veterinarian and farmer verbal behaviour coding from herd health consultation data recorded before and after veterinarians’ experience of brief Motivational Interviewing training. (DOCX)

S1 Text Intracoder consistency assessment: method, results and conclusions. (DOCX)
