# Supplementary material for: Veterinary communication can influence farmer Change Talk and can be modified following brief Motivational Interviewing training
Source: PLoS One. 2022 Sep 12;17(9):e0265586. doi: 10.1371/journal.pone.0265586 (PMC9467306; doi:10.1371/journal.pone.0265586)
Supplement: S1 Table — (DOCX) [file pone.0265586.s001.docx]

**S1. A brief description of the 10 verbal behaviours, 4 globals and 6 summary measurements used in the assessment of Motivational Interviewing skills (Moyers et al., 2014).**

| Variable | Interpretation |
| --- | --- |
| FREQUENCY COUNTS OF BEHAVIOUR | |
| *Giving Information* | Gives information, educates, provides feedback, or expresses a professional opinion without persuading, advising, or warning (i.e. does not imply the information is specifically relevant to the client or that the client must act on it) |
| *Persuade* | Overt attempts to change a client’s opinions, attitudes or behaviors using tools such as logic, compelling arguments, self-disclosure, facts, biased information, advice, suggestions, tips, opinions, or solutions to problems |
| *Persuade with Permission* | Emphasis on collaboration or autonomy support while using persuasion |
| *Questions* | Open or closed |
| *Simple Reflections* | Repeats, re-phrases or paraphrases the client’s previous statement adding little or no meaning or emphasis to what the client has said |
| *Complex Reflections* | Repeats, re-phrases or paraphrases the client’s previous statement adding substantial meaning or emphasis to what the client has said |
| *Affirmations* | States something positive about the client’s strengths, efforts, intentions or worth |
| *Seeking Collaboration* | Explicitly attempts to share power or acknowledge the expertise of the client |
| *Emphasise Autonomy* | Highlights a client’s sense of control, freedom of choice or self-direction over change |
| *Confront* | Directly and unambiguously disagreeing, arguing, correcting, shaming, blaming, criticizing, labeling, warning, moralizing, ridiculing or questioning a client’s honesty |
|  |  |
| GLOBAL SCORES (SCALE 1-5) | |
| *Cultivating Change Talk* | Encourages the client’s own language in favor of the change goal and confidence for making that change |
| *Softening Sustain Talk* | Avoids a focus on the reasons against changing or on maintaining the status quo |
| *Partnership* | Conveys an understanding that expertise and wisdom about change reside mostly within the client and actively fosters collaboration and power-sharing |
| *Empathy* | Understands or makes an effort to grasp the client’s perspective and experience. Reflective listening is an important part of this characteristic, but it encompasses *all* attempts made to understand the client and actively communicate this understanding |
| SUMMARY MEASUREMENTS | |
| *Relational* | (Partnership + Empathy)/2 |
| *Technical* | (Cultivating Change Talk + Softening Sustain Talk)/2 |
| *Reflections per Question* | (Simple Reflections + Complex Reflections)/Total Questions |
| *Percent Complex Reflection* | Complex Reflections/(Simple Reflections + Complex Reflections) |
| *Total MI adherent* | Seeking Collaboration + Affirm + Emphasise Autonomy |
| *Total MI inadherent* | Persuade + Confront |

Moyers TB, Manuel JK, Ernst D. Motivational Interviewing Treatment Integrity Code 4.2.1. 2014. Unpublished manual.
