# Supplementary material for: Veterinary communication can influence farmer Change Talk and can be modified following brief Motivational Interviewing training
Source: PLoS One. 2022 Sep 12;17(9):e0265586. doi: 10.1371/journal.pone.0265586 (PMC9467306; doi:10.1371/journal.pone.0265586)
Supplement: S2 Table — (DOCX) [file pone.0265586.s002.docx]

**S2. A brief description of the Change Talk, Sustain Talk and Follow/Neutral language captured through Client Language Assessment in Motivational Interviewing coding system (Miller et al., 2008).**

| **Variable** | **Interpretation** |
| --- | --- |
| **Change talk** | |
| Reason  *Subcode: desire*  *Subcode: ability*  *Subcode: need* | Specific rationale, basis, incentive or justification for making the change |
|  | As above, containing words ‘want’, ‘desire’, ‘like’ or close synonym |
|  | As above, containing words ‘can’, ‘possible’, ‘willpower’ or close synonym |
|  | As above, containing words ‘need’ or ‘must’ or close synonym |
| Taking steps | Concrete action(s) towards the change |
| Commitment | Agreement, intention or obligation towards the change |
| Other | Any language that clearly reflects movement towards the change that is not captured by the other categories |
| **Neutral** | |
| Follow/neutral | No indication of client inclination either towards or away from the change |
| **Sustain talk** | |
| Reason  *Subcode: desire*  *Subcode: ability*  *Subcode: need* | Specific rationale, basis, incentive or justification away from making the change or to maintain the *status quo*  As above, containing words ‘want’, ‘desire’, ‘like’ or close synonym  As above, containing words ‘can’, ‘possible’, ‘willpower’ or close synonym  As above, containing the words ‘need’ or ‘must’ or close synonym |
| Taking steps | Concrete actions away from the change or to maintain the *status quo* |
| Commitment | Agreement, intention or obligation away from the change or to maintain the *status quo* |
| Other | Any language that clearly reflects movement away from the change or to maintain the *status quo* that is not captured by the other categories |

*Miller WR, Moyers TB, Manuel JK, Christopher P, Amrhein P. Revision for Client Language Coding: MISC 2.1 Client Language Assessment in Motivational Interviewing (CLAMI) Segment. 2008.*
