# Supplementary material for: Veterinary communication can influence farmer Change Talk and can be modified following brief Motivational Interviewing training
Source: PLoS One. 2022 Sep 12;17(9):e0265586. doi: 10.1371/journal.pone.0265586 (PMC9467306; doi:10.1371/journal.pone.0265586)
Supplement: S3 Table — (DOCX) [file pone.0265586.s003.docx]

**S3. ‘Study-irrelevant’ verbal behaviour codes constructed for analysis of veterinary herd health consultations.**

| **Study-irrelevant behaviour** | **Brief description** |
| --- | --- |
| Veterinarian/farmer irrelevant | Verbal behaviour that offers no insight or influence on the advisory topic under discussion (e.g. discourse relating to moving cows for examination) |
| Veterinarian/farmer talk to cow(s) | Any cow-directed verbal behaviour |
| Third party | Any uninvolved/third party interrupting the consultation |
| Veterinarian/farmer cannot hear content | Verbal behaviour is impossible to code due to background noise and/or recording quality |
| Moving cow(s) | Consultation disrupted by one or both parties moving a cow or cows |
| Veterinarian/farmer communication pause | Any break in veterinarian or farmer discourse >3 seconds (e.g. when veterinarian is examining a cow) |
