# Supplementary material for: Veterinary communication can influence farmer Change Talk and can be modified following brief Motivational Interviewing training
Source: PLoS One. 2022 Sep 12;17(9):e0265586. doi: 10.1371/journal.pone.0265586 (PMC9467306; doi:10.1371/journal.pone.0265586)
Supplement: S4 Table — (DOCX) [file pone.0265586.s004.docx]

**S4. Consultations (n=31) submitted by veterinarians (n=14) representing a ‘change for the benefit of herd health’ either before or after a Motivational Interviewing training experience.**

| Topic | Number of consultations | Consultation | Areas of change discussed with potential for improvement |
| --- | --- | --- | --- |
| Calf health | 9 | 1 | Weaning, feeding, temperature maintenance, housing processes, outdoor pasture allocation/management |
|  |  | 2 | Reducing pneumonia- management to avoid, feeding equipment, housing |
|  |  | 3 | Colostrum feeding practices |
|  |  | 4 | Calf hutches, calf jackets, removing calves from dam, colostrum feeding/testing |
|  |  | 5 | Feeding/weaning, colostrum practice, vaccination protocols, ear tagging protocols (for bovine viral diarrhea; BVD), husbandry- cleaning, bedding, lime, buying in protocols |
|  |  | 6 | Reducing scour, reducing pneumonia |
|  |  | 7 | Colostrum feeding and harvesting, scour prevention, pneumonia prevention, use of dump milk/milk powder, teat management/replacement, nose to nose contact- hurdle use, intranasal vaccination, calving yard, calf hutches, dump milk process/cluster cleanliness |
|  |  | 8 | Scour prevention and treatment, feeding |
|  |  | 9 | Calf scour |
| Herd health  (>one topic area) | 6 | 1 | Reducing mastitis: tackling surge of cases in spring via use of wipes in parlour, sampling and testing protocols  Improving fertility: improving calving patterns, calving interval, 100-day in-calf rate, first service submission rate, return service submission rate, heat detection at pasture (summer) |
|  |  | 2 | Calf and heifer health: housing, feeding reducing pneumonia, gene testing |
|  |  | 3 | Fertility: improving calving intervals, 100-day in-calf rate, first service submission rate, return service submission rate, cow body condition score  Youngstock condition (vaccination, cleaning routines) |
|  |  | 4 | Mastitis: reducing cell count  Herd nutrition  Calf health: bedding  Fertility: calving interval, cows in calf  Vaccination: BVD  Genomics testing |
|  |  | 5 | Heifer health: worming protocols  Housing: scraping protocol, cubicle management  Lameness |
|  |  | 6 | Early lactation: nutrition  Transition cows: provision of feeding opportunities and space |
| Mastitis | 6 | 1 | Reducing subclinical levels of mastitis within the herd, addressing ‘problem cows’ |
|  |  | 2 | Reducing milk fever/ketosis in fresh calvers at turn out, bedding management, milking regime, tubing in the dry period, testing protocols, grazing, cell count management |
|  |  | 3 | Housing, data and paperwork management, cow identification and management |
|  |  | 4 | Use of the Dairy Co. Mastitis Control Plan, drying off of cows |
|  |  | 5 | Addressing teat damage, data recording and interpretation |
|  |  | 6 | Tubing cleanliness, milking cleanliness (wiping, dipping), cubicle management (liming) |
| Lameness | 3 | 1 | White line disease, sole ulcers, trimming, blocking, use of non-steroidal anti-inflammatory drugs, foot baths |
|  |  | 2 | Facilitating early detection and treatment |
|  |  | 3 | Facilitating early detection and treatment |
| Johne’s | 2 | 1 | Culling protocol, calf pen cleaning protocols, testing processes |
|  |  | 2 | Herd management, testing processes |
| Fertility/ breeding | 2 | 1 | Use of progesterone releasing intravaginal devices, use of Estrumate (prostaglandin analogue: control time of oestrus) use and decision making, serving protocols, ‘washing out’ (intrauterine cephapirin) |
|  |  | 2 | Enhancing profitable lifetime index: traits: stature, fertility, milk solids, milk kilos, life span  Goals for serving |
| Heifer health and welfare | 1 | 1 | Reviewing: processes of introduction to system (parlour, cubicles, feed barriers) and milking herd |
| Dry cow health and welfare | 1 | 1 | Nutrition, fertility, breeding protocols (part flying herd), transition cow period |
| Nutrition | 1 | 1 | Supplementing for copper deficiency, adjusting selenium, use of bolus treatment, improving mineral levels |
