# Supplementary material for: Veterinary communication can influence farmer Change Talk and can be modified following brief Motivational Interviewing training
Source: PLoS One. 2022 Sep 12;17(9):e0265586. doi: 10.1371/journal.pone.0265586 (PMC9467306; doi:10.1371/journal.pone.0265586)
Supplement: S5 Table — (DOCX) [file pone.0265586.s005.docx]

**S5. A summary of veterinarian and farmer verbal behaviour coding from herd health consultation data recorded before and after veterinarians’ experience of brief Motivational Interviewing training.**

| Participant | MI- adherent | MI -inadherent | Other | Question | Reflection | Change Talk | Follow/  Neutral | Sustain Talk | Reflections per Question | Percent Complex Reflections | Relational | Technical | Proportion farmer talk |
| --- | --- | --- | --- | --- | --- | --- | --- | --- | --- | --- | --- | --- | --- |
| PRE-TRAINING | | | | | | | | | | | | | |
| 1 | 3.00 | 3.00 | 17.00 | 5.00 | 4.00 | 11.00 | 29.00 | 3.00 | 0.80 | 50.00 | 2.50 | 3.00 | 53.87 |
| 2 | 0.71 | 3.60 | 14.87 | 6.53 | 2.20 | 6.55 | 12.68 | 0.71 | 0.34 | 0.00 | 1.00 | 2.50 | 33.58 |
| 3 | 0.00 | 18.78 | 34.25 | 24.31 | 6.63 | 12.15 | 44.20 | 13.26 | 0.27 | 0.00 | 1.50 | 2.50 | 32.08 |
| 4 | 0.00 | 8.51 | 29.79 | 42.55 | 0.00 | 8.51 | 46.81 | 0.00 | 0.00 | 0.00 | 1.00 | 2.50 | 42.22 |
| 5 | 2.00 | 18.00 | 36.00 | 26.00 | 8.00 | 15.00 | 46.00 | 5.00 | 0.31 | 25.00 | 1.50 | 2.50 | 34.36 |
| 6 | 2.57 | 16.68 | 53.90 | 38.50 | 11.55 | 10.27 | 48.77 | 8.98 | 0.30 | 0.00 | 1.00 | 2.50 | 33.51 |
| 7 | 0.00 | 5.26 | 29.78 | 24.53 | 5.26 | 14.01 | 35.04 | 1.75 | 0.21 | 33.33 | 1.50 | 2.50 | 33.50 |
| 8 | 0.00 | 17.30 | 45.61 | 20.45 | 12.58 | 14.15 | 48.75 | 3.15 | 0.62 | 0.00 | 1.00 | 2.50 | 22.71 |
| 9 | 0.00 | 11.67 | 23.35 | 21.01 | 18.68 | 18.68 | 44.36 | 14.01 | 0.89 | 25.00 | 3.00 | 3.00 | 27.95 |
| 10 | 12.00 | 11.00 | 31.00 | 21.00 | 6.00 | 11.00 | 34.00 | 7.00 | 0.29 | 16.67 | 3.50 | 3.00 | 66.57 |
| 11 | 0.00 | 14.00 | 47.00 | 6.00 | 4.00 | 7.00 | 46.00 | 0.00 | 0.67 | 0.00 | 1.50 | 2.50 | 25.06 |
| 12 | 2.00 | 7.00 | 34.00 | 42.00 | 6.00 | 11.00 | 45.00 | 11.00 | 0.14 | 33.33 | 1.50 | 2.50 | 35.25 |
| 13 | 0.00 | 0.00 | 58.00 | 5.00 | 3.00 | 2.00 | 57.00 | 1.00 | 0.60 | 0.00 | 2.00 | 1.50 | 24.81 |
| 14 | 0.00 | 16.72 | 29.27 | 32.06 | 6.97 | 16.72 | 45.99 | 6.97 | 0.22 | 40.00 | 2.00 | 2.50 | 16.91 |
| Mean | 1.59 | 10.82 | 34.56 | 22.50 | 6.78 | 11.29 | 41.69 | 5.42 | 0.40 | 15.95 | 1.75 | 2.54 | 34.46 |
| POST-TRAINING | | | | | | | | | | | | | |
| 1 | 1.00 | 2.00 | 27.00 | 23.00 | 3.00 | 10.00 | 37.00 | 2.00 | 0.13 | 0.00 | 2.50 | 2.50 | 48.44 |
| 2 | 0.00 | 2.60 | 26.97 | 21.57 | 7.52 | 8.42 | 24.47 | 2.00 | 0.35 | 66.67 | 3.50 | 3.50 | 34.53 |
| 3 | 7.32 | 3.66 | 25.61 | 23.78 | 20.12 | 14.63 | 31.10 | 5.49 | 0.85 | 54.55 | 4.00 | 4.00 | 38.66 |
| 4 | 1.00 | 6.00 | 24.00 | 19.00 | 3.00 | 2.00 | 40.00 | 0.00 | 0.16 | 33.33 | 1.00 | 2.50 | 57.29 |
| 5 | 0.00 | 7.00 | 12.00 | 16.00 | 22.00 | 20.00 | 36.00 | 14.00 | 1.38 | 54.55 | 4.50 | 4.50 | 65.39 |
| 6 | 0.00 | 7.13 | 66.98 | 9.98 | 11.40 | 14.25 | 38.48 | 9.98 | 1.14 | 0.00 | 1.00 | 2.50 | 37.38 |
| 7 | 4.00 | 0.00 | 17.00 | 16.00 | 20.00 | 40.00 | 26.00 | 3.00 | 1.25 | 50.00 | 5.00 | 4.50 | 53.64 |
| 8 | 0.00 | 12.61 | 32.77 | 34.03 | 27.73 | 21.43 | 57.98 | 8.82 | 0.81 | 9.09 | 2.50 | 2.50 | 48.08 |
| 9 | 5.91 | 5.91 | 25.12 | 32.51 | 13.30 | 35.47 | 29.56 | 1.48 | 0.41 | 0.00 | 4.50 | 4.00 | 44.02 |
| 10 | 3.00 | 3.00 | 17.00 | 39.00 | 17.00 | 38.00 | 47.00 | 9.00 | 0.44 | 70.59 | 5.00 | 4.50 | 59.80 |
| 11 | 2.67 | 1.34 | 29.40 | 28.06 | 20.04 | 37.42 | 44.10 | 6.68 | 0.71 | 13.33 | 4.00 | 4.00 | 53.19 |
| 12 | 5.50 | 2.50 | 34.00 | 16.00 | 8.00 | 18.50 | 37.50 | 2.50 | 0.50 | 12.50 | 2.75 | 3.25 | 44.85 |
| 13 | 4.27 | 4.27 | 38.43 | 29.89 | 29.89 | 29.89 | 76.87 | 8.54 | 1.00 | 42.86 | 3.00 | 4.00 | 61.91 |
| 14 | 4.81 | 10.82 | 14.43 | 10.82 | 12.02 | 32.46 | 10.82 | 9.62 | 1.11 | 50.00 | 4.00 | 4.50 | 43.58 |
| Mean | 2.82 | 4.92 | 27.91 | 22.83 | 15.36 | 23.03 | 38.35 | 5.94 | 0.73 | 32.68 | 3.38 | 3.63 | 49.34 |
| PRE- POST BRIEF MI TRAINING COMPARISON | | | | | | | | | | | | | |
| *p*-value | 0.5 | 0.001 | 0.04 | 0.91 | 0.01 | 0.02 | 0.42 | 0.72 | 0.04 | 0.08 | 0.0001 | 0.01 | 0.01 |

MI = Motivational Interviewing; Yellow shading = meets global *and* verbal behaviour ‘fair’ competency; Green shading = meets global ‘fair’ competency (Moyers et al. 2014); MI-adherent = Emphasise Autonomy + Seek Collaboration + Affirm; MI-inadherent = Persuade + Confront; Other = Persuade with Permission + Give Information + Structuring Statements; Question = Open question + Closed question; Reflection = Simple Reflection + Complex Reflection; Change Talk = all subcodes (Reason + Commitment + Taking Steps + Other); Sustain Talk = all subcodes (Reason + Commitment + Taking Steps + Other); Reflections per Question = (Simple Reflection + Complex Reflection)/Total Questions; Percent Complex Reflection = Complex Reflection/ (Complex Reflection + Simple Reflection); Relational = Partnership + Empathy/2; Technical = Cultivating Change Talk + Softening Sustain Talk/2
