# Supplementary material for: Veterinary communication can influence farmer Change Talk and can be modified following brief Motivational Interviewing training
Source: PLoS One. 2022 Sep 12;17(9):e0265586. doi: 10.1371/journal.pone.0265586 (PMC9467306; doi:10.1371/journal.pone.0265586)
Supplement: S1 Text — (DOCX) [file pone.0265586.s006.docx]

**S6. Intracoder consistency assessment: method, results and conclusions.**

***Method***

For completeness and because all coding was completed by the first author (AB), coding consistency was tested in advance of further statistical analysis statistically in two ways. First, as a measure of point-by-point reliability for sequentially coded data, an event-based Kappa coefficient was calculated using GSEQ 5.1 (1) based on a sub-sample of randomly selected double-coded consultation files (n=4, >10% total observation time, 52 minutes of 490 minutes total). Codes were matched utterance by utterance using an alignment algorithm implemented in GSEQ. Second, data on veterinarian global scores were analysed to determine if theoretically meaningful associations existed in coding attributions between veterinarian verbal behaviours (MI-adherent, MI-inadherant, Other, Reflection, Question) and global measures (Relational global, Technical global) using SPSS Statistics 23 (IBM Corp., Armonk, NY). As global data were non-parametric, a Spearman’s rank correlation was performed.

***Results***

*Sequential coding consistency*: Point-by-point reliability for overall categories was ‘almost perfect’ (Landis and Koch, 1977) at (K(E)=0.81) indicating strong agreement across repeated coding observations in the consultation sub-sample.

*Relational global score and veterinarian verbal behaviour:* analysis indicated a statistically significant positive association between a veterinarian’s Relational global and the use of Reflection *(p<0.0005),* Reflection to Question ratio (*p=0.01*) and percent Complex Reflections (*p=0.001*), as well as statistically significant negative association between a veterinarian’s Relational global and MI-inadherent (*p=0.04)* and Other (*p=0.02)* verbal behaviour.

*Relational global score and farmer verbal behaviour:* analysis indicated a statistically significant positive association between a veterinarian’s Relational global and farmer use of Change Talk *(p=0.001).*

*Technical global score and veterinarian verbal behaviour*: analysis indicated statistically significant positive associations to the use of Reflection *(p<0.0005),* Reflection to Question ratio (*p=0.02*) and percent Complex Reflections (*p<0.0005*), and statistically significant negative association to ‘Other’ verbal behaviour (*p=0.003)* and the proportion of consultation time attributed to veterinarian speech (*p=0.05).* A negative trend was seen in the association between a veterinarian’s Technical global and MI-inadherent behaviour (*p=0.08).*

*Technical global score and farmer verbal behaviour:* analysis indicated a statistically significant positive association between a veterinarian’s Technical global and farmer use of Change Talk *(p=0.001)* and a statistically significant negative association between a veterinarian’s Technical global and farmer use of Sustain Talk *(p=0.04).*

***Conclusions***

Tests of coding consistency indicated that the coder (AB) showed excellent intra-coder agreement, indicating consistency in code attribution throughout the data analysis process. Both the MITI (Moyers et al. 2014) and CLAMI (Miller et al. 2008) codes evidenced theoretically meaningful associations. For example, Relational global scores were positively correlated with Reflection use (*p<*0.0005*)*; the Empathy global of this Relational element attempts to capture ‘*the extent to which the clinician understands or makes an attempt to grasp the client’s perspective and experience... reflective listening is an important part of this characteristic’* (Moyers et al. 2014). Additionally, Technical global scores were positively correlated with Change Talk (*p*=0.001*)* and negatively correlated with Sustain Talk (*p=0.04).* This Technical global represents Cultivating Change Talk, seeking to capture *‘the extent to which the clinician actively encourages the client’s language about change’*, and Softening Sustain Talk, seeking to capture ‘*the extent the clinician avoids a focus on the reasons against changing’* (Moyers et al. 2014). These tests suggest that the coding in this feasibility study was likely to be theoretically sound and reliable across multiple coding sessions.
